# Exposure to carbon monoxide and particulate matter among cassava grits processors in the middle belt of Ghana: a cross-sectional study

**DOI:** 10.11604/pamj.2020.37.181.18489

**Published:** 2020-10-27

**Authors:** Omolola Oyinkan Adeshina, Kwaku Poku Asante, Kenneth Ayuurebobi Ae-Ngibise, Ellen Abrafi Boamah, Oscar Agyei, Reggie Quansah

**Affiliations:** 1 Public Health, Environments and Society, London School of Hygiene and Tropical Medicine, London, United Kingdom,; 2 Public Health, Kintampo Health Research Centre, Kintampo - North Municipality, Ghana,; 3 Public Health, University of Ghana, Accra, Ghana

**Keywords:** Pollution, exposure, cookstove, Ghana

## Abstract

**Introduction:**

exposure to smoke from biomass combustion during economic activities is a major health risk. One of such commercial activities that use biomass fuel is gari (cassava grits) processing. Cassava grits is a staple food produced from grated and fermented cassava. Several studies have depicted exposure to carbon monoxide (CO) and particulate matter (PM_2.5_) at the household level and fewer studies on small-scale industries such as the aforementioned one.

**Methods:**

a cross-sectional study was conducted among 17 cassava grits processors (CGPs) using Lascar CO monitors for 24 hours and micro personal exposure monitoring devices for 72 hours, in the Kintampo South District of Ghana. CGPs were monitored during working hours and off-working hours. Two focus groups were conducted among CGPs and five in-depth interviews among community gatekeepers.

**Results:**

CGPs were exposed to high CO and PM_2.5_ levels during working hours from 6:00 AM - 5:00 PM and off-working hours from 5:00 PM - 5:59 AM. CGPs, community gatekeepers shared different opinions on health effects of biomass fuel use.

**Conclusion:**

traditional cookstoves are used due to the liquefied petroleum gas (LPG) cost, the quantity and the quality of cassava grits from biomass fuel. This activity exposes CGPs to CO and PM_2.5_ concentrations above the 14 ppm safe levels recommended by the World Health Organisation.

## Introduction

Approximately, 3 billion people worldwide, especially, in low and middle-income countries rely on biomass fuel which is easily accessible as their primary source of energy for small-scale industries and cooking in households [1-3]. According to the Sustainable Development Goals (SDGs) 3 and 7 [4,5], affordable and clean energy is fundamental to the health and well-being of not only sustaining an economy but its environment. This is likely to be achieved when there is equity, access and quality for all [5].

Emissions of the inefficient combustion of biomass from these small-scale industries and households affect the air quality in the local environment and homes. This subsequently leads to health problems such as chronic obstructive pulmonary disease (COPD) and respiratory infections [6,7]. In West Africa and Ghana, small-scale industries such as fish smoking, charcoal burning and cassava grits processing emit these gases during these productions. For instance, cassava grits is a staple food which, contributes significantly to food security in the Bono East region [8] with Ghana among the top six producers of cassava in the world [9].

Furthermore, cassava grits processors (CGPs) are mainly women. They are also the primary users of the biomass cookstoves [10] which is used in the final roasting stage of cassava grits. Consequently, they are exposed and more at risk to high levels of carbon monoxide (CO) and particulate matter (PM_2.5_) [11-13]. They spend an average of 11 hours per day exposed to these pollutants. Short and long-term exposures to these pollutants have been linked to reduced life expectancy [11,14].

Exposure to biomass smoke during cassava grits processing differs from that of pollutants emitted from indoor biomass smoke, with regards to the intensity of smoke, exposure frequency and the duration of operating the biomass cookstove. The aim of this study was to investigate exposure CO and PM_2.5_ among CGPs and to assess participants´ knowledge and perception of health and environmental effects of the cassava grits industry in the Kintampo South District, Ghana.

## Methods

**Study area:** Kintampo South District is a district in the Bono East region of Ghana and was formerly one of the 27 districts in the Brong Ahafo region of Ghana [15]. It is situated in the central belt of Ghana with an area of almost 1774.85 km^2^ and a population of approximately 61,716. The area is multi-ethnic but Akans form the majority and their language “Twi” is spoken and understood by majority of the population. About 63.0% of the population are farmers, labourers or domestic workers. Almost 31.1% of the population are in commerce, industry and services [16]. About 80% of the community use biomass fuel as their primary household fuel. The district environmental health department is responsible for implementing the Ghana government policies on air pollution. This research took place in the Kintampo South District that hosts one of the largest cassava grits processing sites in the Bono East region of Ghana.

**Study participants:** there are about 57 CGPs in the Kintampo South district. These CGPs process cassava grits at the roasting stage under an open shed after the cassava crops have undergone the following stages of harvesting, peeling and grating, fermentation [11]. Twenty-two CGPs were met but 17 were recruited; 5 CGPs did not consent. Only CGPs at the roasting stage were recruited, who had at least one-year experience. Selection of CGPs at the roasting stage was the primary focus because of its biomass fuel use. CGPs were purposively selected based on number of years in the cassava grits industry. In addition to the 17 CGPs, 5 gatekeepers were also purposively selected. These gatekeepers were the go-between for the study between the cassava grits industry and district officials in the Kintampo South district. The gatekeepers ensured access to the study area and participants.

**Study design:** we conducted a cross-sectional design with a mixed methods approach between June and August 2015. For the qualitative component, two focus group discussions (FGDs), with 7 women in one group and 10 women in the other, were conducted using an FGD guide. Also, in-depth interviews (IDIs) were conducted with each community gatekeeper using an IDI guide. The FGDs and IDIs were conducted in Twi among the CGPs and in English among gatekeepers. The study was conducted using the platform of the Ghana Randomised Air Pollution and Health Study (GRAPHS). GRAPHS is a cluster randomised trial study with a 3-arm intervention: BioLite, liquefied petroleum gas (LPG) and 3-stone cook stove. This study used validated CO exposure assessment protocols of GRAPHS [10]. For the quantitative component, small, lightweight Lascar CO monitors (Lascar Electronics, London, UK) were used to monitor real time personal CO exposures one-minute intervals among randomly selected CGPs (n=17). The women were encouraged to wear the CO monitors clipped to their clothing and close to the upper chest for 24-hr as per the GRAPHS. MicroPEM (Personal Exposure Monitoring device) were used to measure PM_2.5_ exposures on-minute intervals among randomly selected CGPs (n=14). The MicroPEM devices were given to CGPs in a small bag to wear across the chest for 72-hr as per the GRAPHS [10]. The monitor data were downloaded and analysed at Kintampo Health Research Centre in Ghana.

**Analysis:** descriptive statistics were used to summarize the range calculated for CO and PM_2.5_ levels in a specified time period and their average over a 24-hour period. This was performed to examine the differences between CGPs before and after cookstove activity. The monitored quantitative data was downloaded and analysed at Kintampo Health Research Centre (KHRC) in Ghana. The quantitative CO and PM_2.5_ exposure data were analysed using “R” (GNU GPL version 3 “R” software) to determine levels of exposure among women. The QSR Nvivo version 10® qualitative software was used to assess participants´ knowledge and perception of health and environmental effects of the cassava grits industry. The following were assessed: alternative source of fire for CGPs activities, availability of environmental protection policies and enforcement of policies.

**Ethics approval:** the research did not pose any risks to CGPs, community gatekeepers, but was designed to gather information on cook smoke exposure. All research participants consented to be part of the study. Participation in the research was voluntarily. Scientific and Ethical approval was obtained from the Kintampo Health Research Centre Scientific Review Committee and the Institutional Ethics Committee (00011103).

## Results

The CGPs were aged between 19 and 50 years and had either primary or secondary education. Majority of the CGPs had 10 years working experience in the roasting stage of grits processing and all the GPs were females (Table 1). Community gatekeepers were aged between 36 and 78 years and were at least educated up to the tertiary level (Table 2). All participants answered questions on health concerns and traditional cookstove use in the industry. In this study, the CGPs exposure to CO and PM_2.5_ were assessed.

**Table 1:** demographic characteristics of CGPs

Exposure	Frequency (N=17)	%
**Age (years)**		
19-29	5	29.4
30-40	11	64.7
41-51	1	5.9
**Number of years in region**		
0-20	11	64.7
21-41	5	29.4
42-62	1	5.9
**Number of years in industry**		
0-10	15	88.2
11-21	1	5.9
22-32	1	5.9

**Table 2:** demographic characteristics of community gatekeepers

Exposure	Frequency (N=5)	%
**Age (years)**		
19-40	2	40
41-61	2	40
62-82	1	20
**Number of years in region**		
0-20	2	40
21-41	1	20
42-62	1	20
63-83	1	20

**Figure 1:**
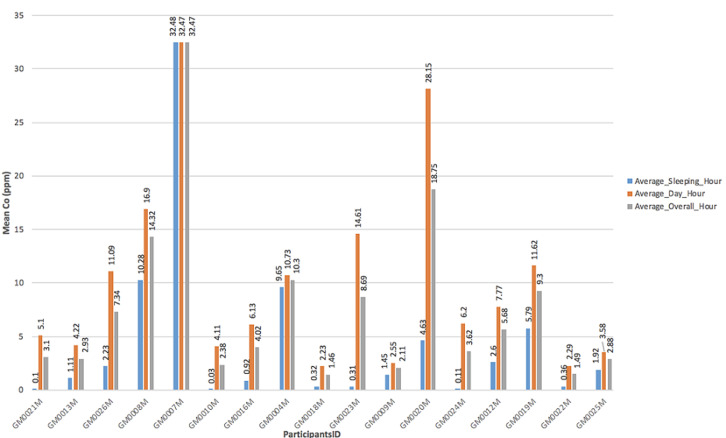
representation of averaged carbon monoxide (CO) at different time range

**CO exposure among CGPs:** the average CO concentration among all participants over a 24-hour period was 7.70 ppm (range 1.46 - 32.47) ppm. During cassava grits processing from 6:00 AM - 5:00 PM (daytime hours), CGPs were exposed to CO levels ranging from 2.23 ppm - 32.47 ppm. Additionally, CGPs were exposed to CO levels ranging from 0.03 ppm - 32.48 ppm when they were either asleep or not performing cassava grits processing from 5:00 PM - 5:59 AM (average night hours). Four out of 17 (23.5%) CGPs were exposed to CO concentrations exceeding the WHO safe levels of 14 ppm [17] (Figure 1).

**Figure 2:**
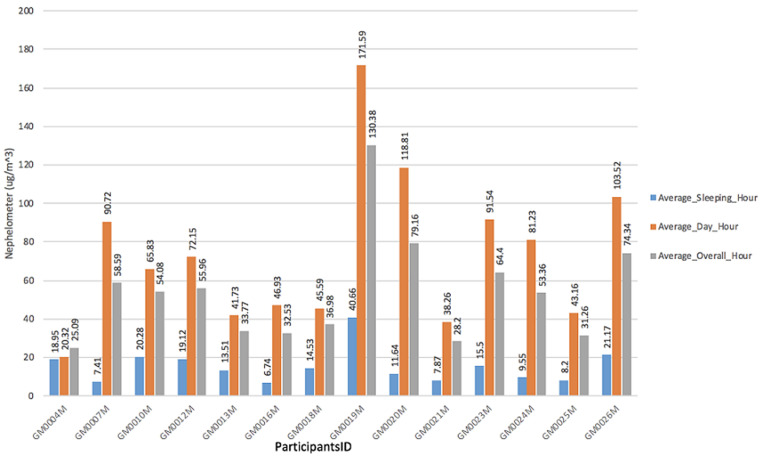
representation of averaged nephelometer at different time range

**PM_2.5_ concentrations among CGPs:** the average PM_2.5_ concentration among all CGPs over a 24-hour period was 54.15μg/m^3^ (range 25.09 - 130.38) μg/m^3^. During cassava grits processing from 6:00 AM - 5:00 PM (daytime hours), CGPs were exposed to PM_2.5_ levels ranging from 20.32 μg/m^3^ - 171.59 μg/m^3^. Additionally, CGPs were exposed to PM_2.5_ levels ranging from 6.74μg/m^3^ - 40.66 μg/m^3^ when they were either asleep or not performing cassava grits processing from 5:00 PM - 5:59 AM (average night hours). Figure 2 shows all CGPs (100%) were exposed to PM_2.5_ concentrations above 25μg/m^3^ air quality interim targets for PM_2.5_ 24-hour concentrations [18] (Figure 2).

**Perception on health effects of pollutants:** community gatekeepers and CGPs recognized continuous exposure to smoke is harmful to the health of CGPs and other members of the community. Women complained of chest pains, eye irritations, and breathing problems: “I also know that the smoke has some effects on the lungs and this is a general problem since the smoke is in the air. Gradually, as time goes on we will have that effect” (IDI, gatekeeper 2). “Some people complain that, their eyes swell, they hardly sleep in the night, they cough and sometimes serious heart aches. I also develop bodily pains” (FGD, woman with 5 years´ experience in cassava grits processing).

However, some other CGPs suggested long-term exposure to pollutants had no adverse health effects on their unborn babies. The quotes below represent the opinion of participants: “since I came to this community for a long time now, we have been frying cassava grits and most pregnant women do fry it. None of them have however had any unfavourably birth outcome” (FGD, woman with 20 years in cassava grits processing). “For me, when I get pregnant, I continue to fry gari throughout my pregnancy till delivery and nothing happens to the children. I have also not received any complain from the doctor about the children which is caused by the smoke” (FGD, woman with 5 years´ experience in cassava grits processing). Some women indicated they discontinued cassava grits processing during pregnancy because they felt warmer by the fireside when processing cassava grits: “when you are pregnant your body feels hotter than someone who would stand by a stove to fry cassava grits. So I stop the cassava grits business during pregnancy” (FGD, woman with 4 years´ experience in cassava grits processing).

**Preference for traditional cookstove:** though firewood was said to emit a lot of smoke, it was the main source of fuel used for processing cassava grits. Respondents suggested that it yielded a larger quantity and quality of cassava grits. Moreover, respondents stated the cost of firewood is lower compared to that of LPG: “a bag of charcoal or gas cooker will emit less smoke, but we will not get the same quality or quantity of cassava grits as this firewood stove. This is cheaper for us” (FGD, woman with 30 years´ in cassava grits processing). “When you want to use charcoal or gas to fry gari and you buy ten bags of charcoal for example, it cannot fry one pan of gari. We will spend more on charcoal or gas” (FGD, woman with 20 years´ in cassava grits processing).

Stacks of wood were observed in the study communities. CGPs retrieved the stacks of wood used for the cook stoves from nearby farmlands. This according to respondents is easily available compared to the LPG which they may have to travel to the nearest town to purchase. “I do not know of any other source of fuel that can be used to fry gari aside the firewood we go out and source for near us” (FGD, woman with 4 years´ experience in cassava grits processing). Gatekeepers have identified over dependence on firewood as a reason for deteriorated farmlands in the study area and its potential impact on sustainability of their natural resources.

**Measures to control pollutants:** this industry in the community has no separate site for its cassava grits processing activities, thus there is no control of the pollutants. A CGP further explained: “we do not really have anything to protect ourselves from the smoke. If it were some body pains then you can buy some drugs to take, but for the smoke we do not have anything to control it” (FGD, woman with 10 years in cassava grits processing). Other measures were proposed by gatekeepers to control the amount of smoke during cassava grit production include use of chimney stoves. A chimney model stove was used on trial within the cassava grits processing industry within the study area. “Recently the district assembly, through business and advisory centre brought some resource people to train us on how to make a chimney on top of the stove/oven used for the cassava grits production” (IDI, gatekeeper 2). “This will serve as an outlet for the smoke so that it does not affect the women who are involved in cassava grits processing unlike what is currently use” (IDI, gatekeeper 3). Gatekeepers have discussed solutions that can be implemented in the community to control the air pollution. Thus far, no other practical measures have been provided to control pollutants.

## Discussion

Individuals using biomass fuel for small-scale activities and household cooking are exposed to high levels of CO and PM_2.5_. The current research explored air pollution exposure from small-scale activities that women engage in while using traditional cookstoves. There was an association between biomass fuel use and CO and PM_2.5_ exposure among women who use biomass fuel for gari processing. Exposure to the biomass fuel peaked during the day when the cassava grits activity was intense compared to night times.

CGPs were aware of alternative sources of fuel such as LPG but their choice of biomass fuel was influenced by cost, perception of higher yield of larger quantity and quality of cassava grits; as well as availability of clean fuels. The CGPs did not have funds to purchase the LPG and did not think they would get value for their money with the purchase of an LPG. With the traditional cookstoves, they were able to make more turnovers and profit in less time than with the LPG. It will beneficial to use the LPG than the traditional cookstoves but CGPs are of the opinion of a negative return on the quantity and quality of cassava grits produced.

The finding of an association between biomass fuel use and CO and PM_2.5_ exposure among women who use biomass fuel for gari processing is similar to what was found in cases in the energy for sustainable development on liquefied petroleum gas use and clean cook stoves in several countries in sub-Saharan Africa (SSA). For example, in Ghana´s rural LPG case, a significant difference between the three stone fire stove and CO exposure was found [19,20]. In Cameroon, the “masterplan” case indicated large, statistically significant differences between LPG and biomass fuel use, including high levels of PM_2.5_ with prolonged biomass fuel use [21,22]. Results from both cases indicate similar health findings with this current study. However, the reverse was the case with LPG use and CO exposure in both cases.

Additionally, individuals were exposed to CO concentrations higher than 14 ppm that puts humans at risk of developing various ailments including, but not limited to heartaches, cardiovascular diseases, acute respiratory infection, muscle weakness with long-term exposure [17]. Similarly, exposure to high PM_2.5_ concentrations leads to significant health problems with long-term exposure [18]. This risk is not limited only to those involved in the cassava grits processing. It is likely that other community members are also at risk of diseases from CO and PM_2.5_ exposure since these pollutants were also high in the evening when the study participants were at home with other community members. All of which can hinder a country from achieving SDGs 3 and 7: “good health and well-being and access to affordable and clean energy” [4,5].

The health risks of pollutants were well known and experienced by CGPs and gatekeepers in this study and other similar studies [23-25]. However, they do not have practical solutions in reducing this risk to protect their health while enhancing their livelihood. Instead, CGPs spend long hours in cassava grits processing at the expense of their health. Protection of individuals in the cassava grits industry and those outside this industry from pollutants are urgently required.

## Conclusion

Limited studies have been conducted or published on clean cook stoves used for commercial activities such as the cassava grits processing activities. CGPs are exposed to high levels of CO and PM_2.5_ during the cassava grit processing [26]. It is likely biomass fuel will remain an integral source of energy for food production industries such as the cassava grits industries for years to come. Individuals in other food production industries may also be exposed to similar levels of CO and PM_2.5_ as found in this study [27]. Attention has not been given to the cassava grits industry to combat the emissions of CO and PM_2.5_ from the biomass fuel use [23]. Improved cookstoves such as LPGs should be provided to CGPs in this industry to significantly reduce the high concentrations of CO and PM_2.5_. It is also important to educate both the CGPs and gatekeepers on the impact of air pollution on an individuals´ health. The inclusion of CGPs to the research agenda would be relevant to Ghana and the rest of SSA where most subsistence daily activities impact the environment [28].

### What is known about this topic

- Individuals in the cassava grits industry are exposed to high levels of CO and PM_2.5_;
- Limited LPG cook stoves targeted for individuals in the cassava grits industry;
- Few studies conducted on exposure levels emitted from cook stoves in this Industry.

### What this study adds

- Twenty-four-hour CO and 72-hr PM_2.5_ exposure measurements at working and non-working hours in the cassava grits industry;
- CGPs are exposed to CO and PM_2.5_ levels during working hours that are harmful to the health;
- Non-CGPs are also at risk of developing health problems during cassava grits processing hours.
